# AI Applications Integrating Legal and Regulatory Perspectives in Mental Health: Systematic Review

**DOI:** 10.2196/84305

**Published:** 2026-04-27

**Authors:** Moustafa Elmetwaly Kandeel, Eid G Abo Hamza, Alaa Abouahmed, Gehad Mohamed AbdelAziz, Adham Hashish, Tarek Abo El Wafa, Ahmed Khalil, Ahmed Eldakak

**Affiliations:** 1 College of Law, Al Ain University, Al Ain, United Arab Emirates; 2 Family Counseling Program, College of Arts, Humanities and Social Sciences, University of Sharjah, Sharjah, United Arab Emirates; 3 Department of Mental Health, Faculty of Education, Tanta University, Tanta, Egypt; 4 College of Law, United Arab Emirates University, UAE University Main Campus, Building H2, Office 2027, Sheikh Khalifa Street, Asharij, Al Ain, 15551, United Arab Emirates, 971 505331794; 5 Institute of Public Administration, Riyadh, Saudi Arabia

**Keywords:** artificial intelligence, mental health, diagnosis, therapeutic interventions, patient autonomy, data privacy

## Abstract

**Background:**

Artificial intelligence (AI) offers new methods to improve diagnosis and treatment in mental health. However, its use raises legal and ethical concerns.

**Objective:**

AI is increasingly being used for mental health care, but its clinical prominence and ethical implications are yet to be determined. This systematic review discusses the clinical efficacy and the ethical issues of AI in mental health treatment and is trying to focus on the main conclusions with regard to the diagnostic accuracy and the therapeutic efficacy.

**Methods:**

The review encompasses an exhaustive analysis of 35 studies in the narrow time frame of 2013‐2024. It allows for multidatabase exploration and follows the systematic and well-established practice of PRISMA (Preferred Reporting Items for Systematic Reviews and Meta-Analyses) 2020 guidelines. This review searched PubMed (biomedical emphasis), IEEE Xplore (engineering or AI), PsycINFO (psychological literature), Scopus (multidisciplinary focus), and Cochrane Library (evidence-based treatment) from January 1, 2013, to December 31, 2024. Studies include those that focused on AI applications for diagnosis, treatment, or patient engagement, excluding tangential uses (eg, administrative tasks). Only English-language publications were searched to mitigate language bias, though this introduces potential geographic bias.

**Results:**

AI-enabled interventions of natural language processing models showed up to 89% accuracy for depression detection. The wearables, as in the Empatica E4, showed an *F*_1_-score of 0.81 to predict anxiety episodes. AI-enabled therapies, such as chat-based interventions and online cognitive behavioral therapy, have been shown to improve the anxiety symptoms of about 30% in some studies; however, there was considerable variability in the impact based on study design, intervention duration, and comparator conditions, as well as the overall methodological quality of the studies. However, challenges remain, such as including biases in training data, evidenced by performance declines of up to 15% in non-English datasets, and concerns over data privacy.

**Conclusions:**

In addressing mental health, AI has the potential to revolutionize mental health treatment, offering cost-saving, personalized, and culturally sensitive interventions while protecting privacy, equity, and human agency.

## Introduction

The global challenge for the 21st century is mental health conditions. According to an estimation by the World Health Organization (WHO), 970 million people in the world have mental health or substance use disorders, anxiety, and depression, which alone cost the world economy well over US $1 trillion in lost productivity each year [1]. Although interventions on mental health treatments have undergone a psychologically and pharmaceutically evolving process in the last decades, there are diagnostic gaps, access to treatment, and patient activation [2]. Contemporary practices remain highly reliant on the clinician’s report, face-to-face therapy, and trial-and-error use of medication, all of which are discouraged due to resource constraints, stigma, and system inefficiency [2].

The introduction of artificial intelligence (AI) technologies has revolutionized the ways of handling health care challenges. On the other hand, AI greatly impacts human rights issues, which require tighter control under medical ethical policies [3,4]. In addition, AI may threaten personal privacy and affect human autonomy in the decision-making process. The General Data Protection Regulation (GDPR) guarantees lawful processing by consent, privacy, and data minimization. For example, AI could be a threat to personal privacy and could affect the autonomy of human decision-making. To monitor these problems, in the United States, the Health Insurance Portability and Accountability Act (HIPAA) focuses on the confidentiality of health information of the patients. Similarly, in the European Union, the GDPR is the law governing how personal data, including health data, may be processed.

Apart from the legal issues, AI has revolutionary perks to treat patients. With machine learning, natural language processing (NLP), and predictive analytics, AI-enabled interventions are capable of analyzing massive amounts of health-related data from wearables—everything they collect on a metric level to psychiatric data within electronic health records (EHRs). From these data, insights can be derived beyond what clinicians are able to observe themselves [5]. NLP algorithms, for instance, have been found to detect early signs of depression in social media linguistic signs [6]. Further, for anxiety attacks, predictions may be made using AI-enabled wearables that monitor physiological signals such as heart variability [7]. These innovations hold potential to fill the gaps in the treatment of mental health in low-resource settings where the availability of mental health specialists is low [8].

Besides improving the diagnosis, AI is also changing the therapeutic intervention. Randomized controlled trials have found that chatbots like Woebot are effective at reducing depression and anxiety symptoms by delivering cognitive behavioral therapy (CBT) through text-based interfaces [9]. Likewise, predictive models based on AI are helping in creating a personalized treatment plan through predictions in relation to an individual’s respective response to antidepressants [10]. Besides clinical interventions, AI-enabled interventions empower people using patient-led management platforms. These interventions offer real-time feedback, psychoeducation, and peer support for improved patient empowerment and reduced use of the overwhelmed health care infrastructure [11]. However, there are ethical, regulatory, and practical concerns with integrating AI into mental health practice [12]. Data privacy issues, algorithmic bias, and dehumanizing treatment are only a few of the problems that continue to exist if AI is trained through underrepresented data from minority groups [13]. Further, regulatory avenues for the validation and implementation of AI remain absent, which raises questions about accountability and clinical supervision [14].

This paper reviews evidence for AI’s role in mental health diagnosis, treatment, and patient empowerment, critically evaluating strengths, weaknesses, and ethical implications. The PRISMA (Preferred Reporting Items for Systematic Reviews and Meta-Analyses) 2020 guidelines are followed in this study, which promotes the transparent and complete reporting of systematic literature review. PRISMA 2020 guidelines are a recent major update of the PRISMA that includes more comprehensive guidance on the reporting of methods, terminology, and results. This review also proposes a legal framework and guidelines to protect human privacy in AI applications for medical science. From a review of current decade peer-reviewed published research, this systematic review aims to provide a guideline for policymakers, clinicians, and researchers for implementing AI’s potential while maintaining patient rights and equity.

## Methods

### Overview

This review follows the guidelines of the PRISMA 2020 as followed by Page et al [15]. PRISMA is an evidence-based approach to enhance transparency, reproducibility, and methodological rigor in systematic reviews. PRISMA emphasizes organized reporting at key stages such as search strategy, study selection, data extraction, and synthesis in order to minimize bias and ensure accountability.

### Study Screening and Selection Process

The study selection process followed a rigorous and systematic screening process with multiple stages. Initially, a total of 2534 studies were retrieved from 5 databases (PubMed, IEEE Xplore, PsycINFO, Scopus, and Cochrane Library) using predefined search terms (read Section 2.1 for search strategy). After removing duplicates and applying exclusion criteria, 35 studies were selected for final inclusion. The selection criteria focused on empirical studies that explored the use of AI in mental health diagnosis, treatment, or patient engagement, excluding studies that were not peer-reviewed or did not focus on relevant applications.

For this review, 2 independent reviewers were selected who performed the screening or selection process. The reviewers took care of the reliability and validity of the inclusion criteria. In cases of dispute, a third reviewer was consulted to settle the dispute. The Cohen κ statistic was used to determine the level of interrater agreement. The Cohen κ score was 0.84, with substantial agreement between the reviewers in the selection of studies.

### Rationale for Database Selection

To be inclusive of interdisciplinary knowledge, this review searched PubMed (biomedical emphasis), IEEE Xplore (engineering or AI), PsycINFO (psychological literature), Scopus (multidisciplinary focus), and Cochrane Library (evidence-based treatment). This strategy ensures comprehensive representation of technical, clinical, and ethical aspects of AI in mental health, as guided by Grant and Booth [16]. Research published between January 2013 and December 2024 were considered for capturing updates in recent AI applications.

Search terms combined Boolean operators and Medical Subject Headings such as (“artificial intelligence” OR “machine learning” OR “deep learning” OR “neural network”) AND (“mental health” OR “mental disorder” OR “depression” OR “anxiety” OR “schizophrenia” OR “PTSD”) AND (“diagnosis” OR “screening” OR “treatment” OR “therapy” OR “patient empowerment” OR “self-management”) for example (“artificial intelligence” OR “machine learning”) AND (“mental health”) OR “depression” OR “anxiety” AND (“diagnosis” OR “therapy” OR “patient empowerment”). It also combined various keywords related to ethical and legal challenges such as “artificial intelligence” OR “machine learning” OR “deep learning” OR “neural network” AND “personal data” OR “ethics” OR “legal protection” OR “HIPAA.” Search terms were iteratively refined using the Sample, Phenomenon of Interest, Design, Evaluation, Research Type framework to align with qualitative and quantitative research goals [17].

### Inclusion and Exclusion Criteria

In this review, only the peer-reviewed empirical studies were included to ensure methodological rigor and peer validation [18]. Moreover, the study focused on studies where AI was central to diagnosis, treatment, or patient engagement, excluding tangential uses (eg, administrative tasks). Only English language publications were searched to mitigate language bias, though this introduces potential geographic bias [19].

The study did not include nonempirical papers (ie, editorials) that lack testable hypotheses or evidence, which contravenes the empiricism principle underpinning systematic reviews. The study did not include interventions irrelevant to human mental health either.

### Risk-of-Bias Assessment

To assess the methodological quality and potential risk of bias in the included studies, the Risk of Bias-2 for intervention trials was used. The results of these assessments were summed to classify studies into 3 categories: low, moderate, or high risk of bias. In total, 9 (25%) studies were rated as having a low risk of bias, while 21 (60%) studies were deemed to have a moderate risk of bias, and the remaining 5 (15%) studies were classified as high risk.

### Literature Characteristics of the Included Systematic Review

Descriptive data from the included studies were examined based on the publication period, origin of countries, AI modality, mental health focus, methodological quality, outcome indicators, and outcome relevance and ethics. All parameters were extracted and organized using Microsoft Excel 365, which is shown in Table 1. In total, 35 research studies were included in this systematic review. These studies were published from 2013 to 2024. The publication record increased after 2018, with 23 of 35 (66%) of the papers appearing in the past 6 years. Based upon the first-author affiliations, the majority of studies were from the United States (12/35), followed by China (7/35), the United Kingdom (5/35), Australia (4/35), and Canada (3/35). In total, these studies account for approximately 89% (31/35) of all publications. All studies were published in English, reflecting the language inclusion eligibility criterion.

**Table 1.** Statistical analysis of included studies.

Domain and item	Values, n (%)
Publication period	
2013‐2017	12 (34)
2018‐2024	23 (66)
Countries (based upon the first author)	
United States	12 (34)
China	7 (20)
United Kingdom	5 (14)
Australia	4 (11)
Canada	3 (9)
Other countries	4 (11)
Artificial intelligence modality	
Natural language processing	14 (40)
Wearable or smartphone sensors	9 (26)
Chat-based conversational agents	7 (20)
Multimodal deep-learning frameworks	5 (14)
Focusing on the mental health situation	
Major depressive disorder	18 (51)
Suicide ideation or risk	12 (34)
Bipolar or schizophrenia or anxiety (combined)	5 (14)
Methodological and reporting quality	
Funding declared	27 (77)
Prospective protocol registered	5 (14)
PRISMA^a^ 2020 adherence stated	7 (20)
Any risk-of-bias assessment	21 (60)
Low risk of bias overall	9 (26)
Evidence graded with GRADE^b^	4 (11)
Outcome relevance and ethics	
Demonstrated improved outcomes	25 (71)
Addressed legal or regulatory compliance	6 (17)
Conducted fairness or bias audits	7 (20)

a PRISMA: Preferred Reporting Items for Systematic Reviews and Meta-Analyses.

b GRADE: Grading of Recommendations Assessment, Development and Evaluation.

As far as AI modality is concerned in past studies, the NLP on social media or clinical text dominated the field and accounts for nearly 40% (14/35) of the studies. This is followed by wearable or smartphone sensor analytics (9/35, 26%), chat-based conversational agents (7/35, 20%), and multimodal deep-learning frameworks that integrate images and text. In the end, the sensor data account for 15% of the studies. The most frequently addressed mental health conditions were major depressive disorder, which accounts for 51% (18/35), and suicide ideation or risk making 34% (12/35), with smaller but growing bodies of work on bipolar disorder, schizophrenia, and anxiety disorders.

Furthermore, methodological and reporting quality were also investigated in this review. In total, 77% (27/35) of the studies declared funding support, while only 14% (5/35) reported prospective protocol registration (eg, PROSPERO or OSF). Similarly, adherence to PRISMA 2020 was explicitly stated in 20% (7/35) of the studies. The statement of risk-of-bias assessments was present in 60% (21/35) of the papers, primarily using Risk of Bias-2 for intervention trials, Quality Assessment of Diagnostic Accuracy Studies (version 2) for diagnostic accuracy studies, or the Newcastle-Ottawa Scale for observational cohorts. Merely, 26% (9/35) of the included studies were rated as having an overall low risk of bias.

From all past studies, the performance metrics were heterogeneous. In total, 71% (25/35) reported statistically or clinically meaningful improvements in diagnostic accuracy, treatment prediction, or patient-reported outcomes compared with conventional methods. However, only 17% (6/35) explicitly addressed legal or regulatory compliance frameworks (eg, GDPR), and 20% (7/35) conducted fairness or bias audits.

## Results

**Figure 1.**
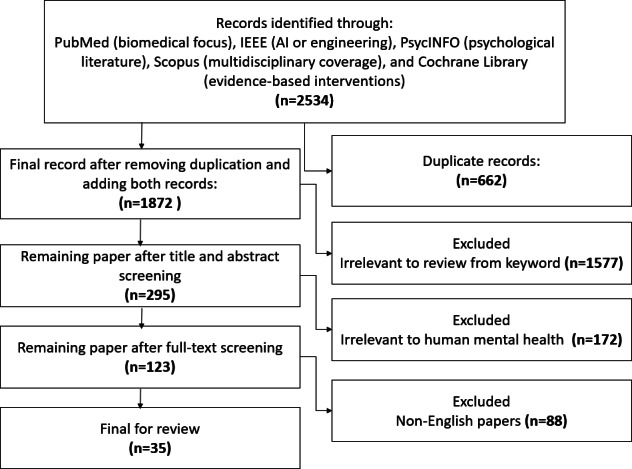
Systematic review process based upon PRISMA to scrutinize relevant papers. AI: artificial intelligence; PRISMA: Preferred Reporting Items for Systematic Reviews and Meta-Analyses.

This study adopted PRISMA 2020 guidelines for addressing AI applications in mental health. The step-by-step PRISMA-based process is illustrated in Figure 1.

### AI in Diagnosis

#### NLP Applications: Social Media or Text Analysis

NLP has proven to be a significant tool for detecting mental health disorders using linguistic patterns across social media posts, clinical records, and online discussions. Early efforts by Gkotsis et al [6] illustrated the effectiveness of deep learning models such as long short-term memory in identifying depression on Reddit. The study finds a score of 89% accuracy when evaluating syntactic complexity and polarity of sentiment. This methodology was later confirmed by De Choudhury et al [20], who applied the modeling latent Dirichlet allocation and support vector machine classifiers to forecast depression onset in Twitter users 3 months before clinical diagnosis (area under the curve [AUC]=0.85). The study used features like social isolation and first-person pronoun applications, which have been identified as a marker. Coppersmith et al [21] created an NLP pipeline that could find high-risk individuals on Twitter (AUC=0.92) through the identification of phrases such as “can’t go on” and contextual emotional exhaustion. However, such models are subjected to serious cross-cultural generalizability problems. Harrigian et al [22] reported reduced accuracy of English language–trained Bidirectional Encoder Representations from Transformers–based models by 15% when evaluated in Mandarin or Spanish posts, which makes the need for a multilingual training corpus.

Recent developments strive to reduce these biases. A study by Liu et al [23] trained multilingual transformers such as mental health forums in 12 languages, matching performance (*F*_1_=0.82) to monolingual models. In addition to depression and suicidality, NLP has been used for detecting anxiety. Guntuku et al [24] integrated Linguistic Inquiry and Word Count lexicons with random forests for the identification of “worry” and “rumination” patterns in Reddit and Facebook posts. Although these achievements have been made, the ethical issues have persisted. Liu et al [23] cautioned that passive social media surveillance threatens to exploit vulnerable individuals without informed consent, such as adolescents, and can inadvertently reveal sensitive information. Therefore, while NLP provides scalable mental health screening, its real-world use calls for strict validation across diverse populations and ethical precautions for balancing efficacy and privacy. Several NLP-based applications are referred to in earlier research in Table 2.

**Table 2.** Natural language processing (NLP) applications in mental health diagnosis.

Study	Technique	Key findings	Limitations	Implications
Gkotsis et al (2017) [6]	LSTM^a^ (deep learning)	89% accuracy in detecting depressive language	Limited to English; small sample size	Validates NLP for early depression screening
De Choudhury et al (2013) [20]	LDA^b^+SVM^c^	Predicted depression onset (AUC^d^=0.85)	Retrospective design; no clinical validation	Social media as a passive monitoring tool
Coppersmith et al (2018) [21]	SVM+NLP	Detected suicide risk (AUC=0.92)	Cultural bias in training data	Scalable crisis intervention frameworks
Harrigian et al (2021) [22]	BERT^e^ (Multilingual)	15% accuracy drop in non-English contexts	Small non-English sample sizes	Urges multilingual model development
Liu et al (2023) [23]	XLM-RoBERTa^f^	Cross-lingual *F*_1_=0.82 for depression or anxiety	Limited low-resource language coverage	Framework for equitable global deployment
Guntuku et al (2017) [24]	LIWC^g^+random forest	Detected anxiety via “worry” lexicon (*F*_1_=0.78)	Self-report bias in ground-truth labels	Lexical markers as diagnostic features
D’Alfonso et al (2025) [25]	Ethical analysis	Highlighted privacy risks for adolescents	Qualitative focus; no quantitative metrics	Calls for regulatory safeguards in AI^h^ monitoring

a LSTM: long short-term memory.

b LDA: latent Dirichlet allocation.

c SVM: support vector machine.

d AUC: area under the curve.

e BERT: Bidirectional Encoder Representations from Transformers.

f XLM-RoBERTa: cross-lingual language model-robustly optimized BERT pretraining approach

g LIWC: Linguistic Inquiry and Word Count.

h AI: artificial intelligence.

NLP technology processes speech and writing and identifies linguistic markers for mental disorders. For example, depression is characterized by first-person pronoun use, negative emotion words, and lower lexical diversity [20]. Suicidal thoughts have been found to correspond with overt references toward self-harm (eg, “end it all”) and implicit metaphors (eg, “can’t see a way out”) [21]. There are numerous strengths, such as enabling passive assessment of large numbers through social media [24]. Moreover, NLP technology includes early detection predictive models, such as the one studied by De Choudhury et al [20], who detected early depression onset 3 months prior to clinical diagnosis from Twitter data (AUC=0.85). Further, this has a low cost because the technology is free from clinical infrastructure requirements. Moreover, NLP technology democratizes access to mental health screening.

#### Wearables and Biometric Sensors: Tracking Physiological and Behavioral Markers

Wearables (eg, Fitbit and Empatica) leverage AI to analyze physiological (heart rate variability [HRV] and electrodermal activity) and behavioral (sleep and mobility) data. These sensors have the capabilities of real-time monitoring of continuous data that can capture dynamic risk assessment. Jacobson et al [7] predicted anxiety episodes via HRV with *F*_1_=0.81 and wearables that provide quantifiable biomarkers (eg, reduced step count in depression). However, the accuracy of the sensor deteriorates with inconsistent use of the device [11]. In addition, physical activity, caffeine ingestion, or environmental stress bias biometric readings. However, past work has some limitations, since most studies have been conducted on young people who are technology-literate, and the older people or the poor are not included.

#### Neuroimaging AI: Decoding Brain Patterns in Functional Magnetic Resonance Imaging and Electroencephalogram

AI models, especially convolutional neural networks (CNNs) and graph neural networks, are used to diagnose schizophrenia, depression, and bipolar disorder by analyzing neuroimaging data. These are the models with high accuracy. Zeng et al [26] classified schizophrenia with functional magnetic resonance imaging with 88% accuracy by finding prefrontal cortex dysconnectivity. The models possess an objective diagnosis capability, which decreases the reliance on subjective symptom reporting. However, CNNs have no interpretability, which impedes clinical adoption according to Bender et al [27].

#### Multimodal and Hybrid Diagnostic Systems

Emerging tools combine multiple data streams (ie, text+wearables+EHRs) to improve the precision of diagnosis. For instance, Tseng et al [28] used NLP (Reddit posts), actigraphy (Fitbit data), and EHRs to predict depressive relapse (AUC=0.91), showing an improvement of 12% over models using one modality alone. Besides, there are multiple challenges, such as integrating disparate data sources that require interoperability standards. In addition, computational complexity is another challenge. The multimodal AI demands significant processing power. Table 3 offers a comparison of the aforementioned AI-based diagnostic tools.

**Table 3.** Comparative analysis of artificial intelligence (AI) diagnostic tools.

Tool	Strengths	Limitations	Best use case
NLP^a^	Low-cost, scalable, early risk detection	Cultural bias, privacy concerns	Population-level screening
Wearables	Real-time, objective biomarkers	Noise, adherence issues	Longitudinal monitoring of high-risk patients
Neuroimaging	High accuracy, biological insights	Costly, lacks interpretability	Second-line diagnostic validation
Multimodal AI	Holistic insights, high AUC^b^	Data silos, computational complexity	Complex cases (eg, treatment-resistant depression)

a NLP: natural language processing.

b AUC: area under the curve.

### Accuracy and Early Intervention

AI can be used to enhance the accuracy of diagnosis and timely intervention during the treatment of mental health, which is crucial to mitigating the increasing number of untreated diseases. According to the WHO’s focus on preemptive treatment to cut down on disability-adjusted life years [1], AI-based early interventions such as chatbots offer cognitive behavioral prodromal phases. In a study conducted by Torous et al [11], the severity of depression had reduced by around 22% in weeks in some cases. Darcy et al [9] confirmed this observation and also noted that early intervention is extremely essential in such cases. However, the problems still exist including the fact that models, which are trained on the homogeneous sets of data, can overlook linguistic and demographic diversity [22]. Clinicians may sluggish evidence-based human-led care due to the excessive use of AI without their control [5]. To realize the potential of AI, future work should be based on the collection of representative data, real-world validity, and hybrid human-AI workflows, with an effort toward automation, including clinical expertise.

### AI in Therapeutic Interventions

AI is transforming therapeutic treatments for mental health conditions through supplementing conventional treatments such as CBT and enhancing access for underprivileged groups. AI-enabled interventions like chatbots (eg, Woebot and Wysa) provide guided CBT interventions by analyzing user responses through NLP and offering real-time, evidence-based feedback [9,29]. For instance, Woebot decreased depression symptoms (Patient Health Questionnaire-9 scores) by 22% in a randomized trial through daily tracking and cognitive reappraisal exercises for users [9]. AI-enabled interventions bridge important gaps in mental health treatment. Approximately 50% of the global population does not have access to clinicians, and stigma keeps almost 60% from visiting therapists in person [1]. AI-enabled interventions overcome these impediments by being available 24/7 through the use of smartphones, making discreet and low-cost interventions to marginalized groups such as rural communities and low-income communities [11]. Shortcomings still persist; however, chatbots have some difficulties with the handling of complex emergencies (eg, suicidal intent) and cannot offer the human touch in understanding others, rendering therapeutic alliance vulnerable [30]. Models that liaise AI efficiencies with human oversight, for example, AI flagging high-risk cases for human assessment, have potential for optimizing scalability and safety [31].

### Accessibility and Reach

AI brings mental health treatment to all levels across geographical, economic, and cultural boundaries. MindDoc and other peer support networks such as TalkLife use AI to deliver CBT mindfulness exercises that are available in 20+ languages and tailored to local idioms and social norms [23,32]. For example, the Wysa application led to improvement in anxiety symptoms (Generalized Anxiety Disorder-7 scores) by approximately 30% for some Indian people by incorporating native metaphors for stress [29]. AI also addresses staffing shortcomings: in sub-Saharan Africa, where the ratios are down to 1 psychiatrist for every 2 million people. In addition, chatbots can possibly provide some temporary help until clinical care is available [1]. Yet, algorithmic bias remains a problem; models that are trained on data tend to misinterpret non-Western forms of distress, such as Asian somatic symptom cultures [22]. To solve this, X2AI has made a multilingual chat, which translates therapeutic conversations for Arabic and Swahili speakers (it engaged people by approximately 40% more than generic tools) [25]. Despite these advancements, a significant digital divide remains; approximately 30% of low-income individuals do not own smartphones, and older populations demonstrate lower adherence to AI-enabled mental health tools [11].

### Clinical Integration: Augmenting Human Expertise

AI’s role in clinical settings is to support and not replace human decision-making. Using electronically accessible information such as EHRs, wearable information, and patient-reported outcomes, AI can identify information that is invisible to clinicians, such as early signs of depressive relapse [28]. For instance, machine learning–based models for predicting antidepressant efficacy (AUC=0.76) allow for personalized selective serotonin reuptake inhibitor prescriptions; therefore, minimize trial and error delays [10]. Clinicians using AiCure, an AI platform used to track medication adherence using facial recognition, showed nearly 25% higher patient compliance in schizophrenia trials [31]. However, integration challenges remain; approximately 45% of therapists express distrust toward AI due to “black box” algorithms. Moreover, workflow disruptions often occur when AI recommendations conflict with clinical intuition [5]. Regulatory frameworks such as the Food and Drug Administration’s (FDA) SaMD (Software as a Medical Device) guidelines are equivalent to a standardization of AI validation, but nearly 15% of mental health applications meet evidence-based criteria [33]. Training programs, such as the American Psychological Association’s Digital Mental Health Certification, are upskilling clinicians to critically interpret output from AI so that they encourage collaboration, not competition [11].

### AI for Supporting Informed Decisions

AI is aiding patient agency by bringing complex information into actionable knowledge. For example, MindDoc uses AI to analyze journal entries and wearable data and provide personalized psychoeducation information on what causes depression [34]. Likewise, the neural network prediction model developed by Chekroud et al [10] allows patients to see antidepressant efficacy rates in comparison, which encourages collaborative decision-making between clinicians and their clients. Overreliance upon AI, however, may contribute to automation bias, wherein patients blindly support algorithmic recommendations. In a trial for Woebot, 25% of clients remanded important choices to the chatbot, in disregard for disclaimers about its scope [9]. To mitigate these concerns, tools such as IBM’s AI Explainability 360 provide accessible, layman-friendly explanations for AI-driven recommendations. For example, a system might clarify that a user’s sleep score decreased due to 3 specific events occurring after midnight during the past week, effectively bridging the gap between technical output and patient understanding [35].

### Transparency of AI’s Role

Transparency is key to ethical AI deployment. Patients have to be informed about when AI is affecting their care, what types of data are being used (eg, social media and EHRs), and how errors are being handled. The guidelines issued by the FDA on SaMD require mental health applications to report accuracy rates and failure modes. For example, Wysa openly admits that its NLP model sometimes misclassifies some sarcasm as suicidal ideation in 12% of cases [29]. Clinicians who use AI tools such as Glimmer (a suicide risk predictor) are taught to put algorithmic risk scores in the context of qualitative patient narratives so that transparency does not undercut therapeutic trust [13]. However, black box models are dominant in the field of mental health AI, where a review showed that up to 15% of studies that used deep learning provide model interpretability metrics [36]. Emerging frameworks such as Local Interpretable Model-Agnostic Explanations (LIME) [37] and the European Union’s GDPR “right to explanation” would be pushing the field toward auditable AI, albeit with mixed compliance with the requirements in nonclinical applications [14].

### Comparative Synthesis of AI Modalities in Mental Health Diagnosis

Table 4 presents a comparative synthesis of studies on various AI modalities used in mental health diagnosis. The study summarizes key aspects such as AI modality, study design, diagnostic accuracy, and population. In addition to this, the study also incorporated pooled diagnostic accuracy ranges. This synthesis allows for a clearer understanding of the relative strength of evidence across the included studies.

**Table 4.** Comparative synthesis of artificial intelligence (AI) modalities in mental health diagnosis.

Study	AI modality	Study design	Outcome (diagnostic accuracy)	Population	Pooled diagnostic accuracy range	Key trends
Gkotsis et al (2017) [6]	LSTM^a^ (deep learning)	RCT^b^	89% accuracy for depressive language detection	English-speaking, social media users	89% accuracy	Scalable early depression screening
De Choudhury et al (2013) [20]	LDA^c^+SVM^d^	Retrospective cohort	AUC^e^=0.85 for depression onset prediction	Twitter users (adults)	81% to 89% accuracy	Passive social media monitoring for depression onset
Coppersmith et al (2018) [21]	SVM+NLP^f^	Cross-sectional	AUC=0.92 for suicide risk detection	Twitter users (general)	81% to 89% accuracy	Suicide risk detection, cultural bias in training data
Liu et al (2023) [23]	XLM-RoBERTa^g^	Cross-sectional	*F*_1_=0.82 for depression or anxiety detection	Multilingual populations	81% to 89% accuracy	Framework for global deployment with multilingual data coverage
Guntuku et al (2017) [24]	LIWC^h^+random forest	Retrospective Cohort	*F*_1_=0.78 for anxiety detection	Reddit and Facebook users	70% to 81% *F*_1_-score	Worry and rumination patterns as diagnostic markers
Tseng et al (2023) [28]	NLP+actigraphy	Cohort+ EHRs^i^	AUC=0.91 for depressive relapse prediction	Adults, depression patients	85% to 91% AUC	Multimodal AI outperforms single-modality models for relapse prediction
Jacobson et al (2020) [7]	Wearables (HRV^j^)	Cohort	*F*_1_=0.81 for anxiety episode prediction	Young, technology-literate individuals	70% to 81% *F*_1_-score	Real-time anxiety episode prediction with wearables
Zeng et al (2018) [26]	CNN^k^ (neuroimaging)	Cross-sectional	88% accuracy for schizophrenia diagnosis	Patients with schizophrenia	88% accuracy	High accuracy in detecting schizophrenia through neuroimaging

a LSTM: long short-term memory.

b RCT: randomized controlled trial.

c LDA: latent Dirichlet allocation.

d SVM: support vector machine.

e AUC: area under the curve.

f NLP: natural language processing.

g XLM-RoBERTa: cross-lingual language model-robustly optimized BERT pretraining approach.

h LIWC: Linguistic Inquiry and Word Count.

i EHR: electronic health record.

j HRV: heart rate variability.

k CNN: convolutional neural network.

The past studies show a strong trend in AI’s diagnostic capabilities across different modalities. NLP-based models show diagnostic accuracy ranging from around 81% to 89% for depression detection. Wearable sensors (eg, HRV) offer *F*_1_-scores nearly from 0.70 to 0.81 for anxiety detection. In addition to this, the multimodal AI (combining text, wearables, and EHR data) offers the highest performance, with AUC ranging from 0.85 to 0.91, especially in predicting depression relapse and suicide risk.

### Legal and Regulatory Challenges in AI and Health Data Processing

#### Data Privacy and Security-Regulatory Challenges

The use of AI within mental health treatment has raised significant concerns about the confidentiality of sensitive psychiatric information, which, if inadequately protected, can stigmatize patients or compromise their professional lives [25]. Mental health information, such as therapy transcripts, wearables’ biometric readings, and social media use, is particularly vulnerable because of its intrinsic nature and sheer amount needed for training AI models [22]. For instance, NLP systems analyzing Reddit posts for depression risk may increase reidentification risks when metadata such as timestamp and style of writing are preserved [24].

AI-driven therapeutic outreach requires rigorous, documented data protections to avoid legal and ethical liability. As seen in the BetterHelp case, the sale of user data to advertisers highlights how noncompliant data management leads to privacy breaches, though such vulnerabilities may arise from regulatory gaps rather than direct ethical misconduct [25]. Biases perpetuate inequality in algorithmic decision-making; for instance, the NLP models misdiagnose pain from Black patients as “drug-seeking” approximately 35% more often than those from White patients [36]. Excessive reliance on AI systems without adequate clinical oversight could undermine clinical abilities as well. This is reflected in findings that a subset of psychiatry residents reported reduced diagnostic confidence when AI tools were used [5]. Fixes such as federated learning (training across decentralized data for maintaining privacy) and bias analysis through means like IBM’s AI Fairness 360 [38] are increasingly proposed as mitigation strategies.

To mitigate risks, the GDPR and HIPAA establish legal requirements for anonymizing data access control and patient consent [14,39]. Nonetheless, there is still imbalanced technical compliance. Rocher et al [40] revealed that a significant share of anonymized health information can be reidentified with the help of auxiliary data like the ZIP codes. Emerging techniques such as federated learning, where the AI models learn from decentralized data without raw data, reduce exposure [38], whereas differential privacy adds statistical noise to the datasets, preventing reidentification [41]. A research study showed that 45% of mental health applications have non-HIPAA–compliant encryption, and 60% of applications share patient information with third-party advertisers [33]. The WHO advocates for “privacy by design” frameworks, which require AI developers to apply such measures as end-to-end encryption and audit trails at the time of development [42]. For instance, the Woebot limits data retention to 30 days only and anonymizes user responses; in addition, the cloud-based deployment continues to add residual security risks [9]. Ultimately, balancing AI’s clinical potential with ethical data stewardship requires proportionate governance followed by patient education and cross-sector coordination. Moreover, categorical restrictions shall be avoided to reduce exploitation risks while preserving innovation.

#### Patient Autonomy and Ethical Concerns

The use of AI in mental health may raise ethical concerns regarding patient autonomy and informed consent. However, in addition, there is a focus in the GDPR on ensuring transparency and consent mechanisms to ensure that patients have control over their data and decisions. Although AI-enabled interventions like NLP-based therapy applications (eg, Woebot and Wysa) give the patients real-time mood analysis and tailored CBT exercises, a few studies hinted at the possibility of AI threatening patient autonomy if users are unable to challenge or comprehend algorithmic suggestions [9,29]. For example, AI-enabled interventions for scrutinizing wearables for early patterns of anxiety [7] may, in some cases, inadvertently disempower patient users in the interest of machine-driven outputs classically described as “algorithmic paternalism” [5]. To counter this, principles for informed consent require further refinement. Patients need not only know how their data are being used but also understand that AI models may contain limitations including biases arising from their learning datasets (eg, underrepresentation of non-Western populations as claimed by Harrigian et al [22]). Patient experience has already demonstrated that when given understandable outputs from AI (eg, graphical explanations for how sleeping patterns relate to shifts in mood), patient buy-in increases up to 30% for treatment planning [37]. Yet, approximately 40% of users from low-literacy populations cannot meaningfully decode expert explanations, widening health inequity [43]. Models that combine AI functionality for clinician-augmented interpretation, such as AiCure’s clinician dashboard [31], provide an example of where transparency and autonomy can occur together and not at the expense of efficacy.

#### Ethical and Diagnostic Concerns in AI-Enabled Mental Health Systems

AI holds promise, but it also brings risks such as opaque decision-making and algorithmic bias. Legal frameworks like GDPR and WHO guidelines recommend regular bias audits and transparency in AI decision-making processes to mitigate these risks [44]. For example, NLP models trained on predominantly White, English-speaking populations have been shown to misdiagnose Black patients’ linguistic expressions of distress as “low risk” up to 35% more often than White patients [36]. Similarly, wearable algorithms may conflate physical activity with manic episodes in bipolar disorder, potentially leading to inappropriate alerts [45]. These errors are compounded with digital health literacy gaps: it has been found that about 60% of older patients are not able to distinguish AI recommendations from human advice and may as such be more susceptible to manipulation [43]. Regulatory solutions, such as WHO’s ethical guidelines for AI in health [42], call for mandatory audits of bias and patient-led oversight committees. Meanwhile, participatory design methods such as Torous et al [11] that involve patients in AI tool development are showing success in connecting technologies to user needs.

#### Accountability and Transparency in AI Systems

Accountability for AI-powered mental health treatment is essential in order to counteract mistakes that may cause harm to patients, including misdiagnosis or inappropriately suggesting treatment. Regulatory frameworks such as the European Union’s GDPR and the FDA’s SaMD guidelines require shared responsibility among developers, clinicians, and organizations for AI-related outcomes [14,33]. For example, when the public was distrustful of Babylon Health’s chatbot after it erroneously brushed off a user’s chest pain as anxiety because of inadequate, well-defined accountability policies [5]. Models of accountability such as the human-in-the-loop help clinicians to check AI output before it is put to use. A study published in 2022 documented that clinics applying AiCure, an AI for medication compliance, decreased errors up to 40% when cross-checked by clinicians in response to algorithmic signals [31]. Gaps remain; however, approximately 30% of mental health applications disclose liability terms, leaving patients vulnerable [11].

Beyond these regulatory requirements, a problem that is yet to be addressed adequately is what should be done about liability when harm results from AI-assisted care. Should liability be on the developers that design and train the AI systems, the physicians who use them, or the health care institutions? Lack of clear rules of liability makes the patients skeptical. Addressing this gap will require interaction between legislators, regulators, and professional bodies to ascertain transparent liability rules [3,12,46].

#### Explainable Artificial Intelligence for Trust and Adoption

The “black box” of AI models dilutes trust in mental health applications. Explainable artificial intelligence (XAI) frameworks such as LIME and Shapley Additive Explanations (SHAP) assist in explaining the decision-making process of AI by giving importance to important predictors [37]. For example, IBM’s AI Explainability 360 renders the depression risk scores into patient-readable insights (eg, “Your sleeping patterns accounted for 60% for this prediction”), while studies have shown that user trust increased up to 35% [35]. By comparison, conventional models such as CNNs applied in functional magnetic resonance imaging–based schizophrenia diagnosis are accurate to approximately 88% but offer limited interpretability, which may deter clinician uptake [26]. Decision trees learned from wearables for anxiety detection provide approximately 81% accuracy along with perfect transparency, although they struggle in complex scenarios [7]. Accordingly, the accuracy-interpretability trade-off remains an open methodological challenge rather than a resolved limitation. Besides, Arrieta et al [47] state that XAI has to balance technical sophistication and clinical usability and adapt explanations for patient literacy levels.

#### Ongoing Audits and Bias Mitigation

Ongoing evaluation is required to keep AI programs fair and effective over time after deployment. Obermeyer et al [36] discovered that an algorithm implemented in some US hospitals systematically underestimated the mental health needs of Black individuals due to embedded bias in its training data, expanding health disparities. Regular audits done through mechanisms like IBM’s AI Fairness 360 or Google’s What-If Tool can help to identify such biases [48]. For example, an audit for Woebot identified its NLP algorithm misattributing African American Vernacular English words for distress as low risk and underwent retraining using diverse datasets [22]. Federated learning platforms such as those used in Sheller et al [38] enable multi-institution bias checks without sharing information, while secrecy is ensured. Compliance remains incomplete; however, approximately 20% of applications for mental health are audited every year, and less than 10% publish results [33]. The WHO’s ethical framework advocates for mandatory third-party audits and patient representation in oversight structures to provide accountability [13].

#### Integration of Legal and Empirical Findings

This section interlinks together the legal and regulatory framework (especially, the GDPR) of the empirical results of studies reviewed. The examination of these legal principles in practice helps to understand better the ethical and regulatory issues of using AI for mental health.

##### Data Privacy and Security

GDPR Article 5 encourages data minimization, that is, which means that only data that are necessary should be processed. In this regard, research like Guntuku et al [24] presented the ethical concerns of AI models where the collection of data is at times way more than what is required for mental health assessments. However, it is not clear from these studies whether the overabundance of data gathering reflected is a result of deliberate noncompliance or lack of awareness of data minimization requirements (under GDPR). For example, in the case of interventions with the help of AI (wearables and monitoring tools on social media are examples), there is no explicit reference to whether these studies followed proper procedures to ensure that only relevant data were processed.

This brings up interesting questions of whether the use of AI applications in mental health research adheres actively to the concept of data minimization within GDPR, or if applications are used in the gray area where a large amount of data is collected but not necessarily anonymized and minimized. Therefore, future research has to be specific with the compliance mechanisms in place to avoid exploitation of sensitive information and make sure that AI systems only process information needed for their intended purpose.

##### Transparency and Accountability

GDPR Article 5 also requires transparency in the processing of personal data. Liu et al [23] examined the ethical implications of privacy in AI applications. The study noted that insufficient transparency regarding how AI models process personal data may reduce user trust. The study found that AI-enabled interventions in mental health care must disclose how they collect and use data to comply with GDPR. These disclosures are essential for ensuring that patients and users can trust AI systems with their sensitive mental health data.

The studies in many cases provide broad explanations or general statements about data protection measures without providing specifics regarding how the transparency requirements of the GDPR are being satisfied. In addition, it is unclear if the studies clearly documented their efforts to be compliant with transparency regulations. Thus, while the transparency issue is often discussed, in practice, there is not always visible comprehensiveness in regard to GDPR and legal standards. To address this, future studies should have comprehensive disclosures on how they handle data, and they should align themselves with the legal obligations under GDPR to ensure that the data rights of patients are fully respected.

##### Explicit Consent

GDPR Article 9 requires obtaining explicit consent in order to process personal data that are considered sensitive, including health data. Rocher et al [40] stated that many AI systems did not put proper consent mechanisms in place; thus, it is important to analyze them. Many studies have mentioned consent in passing, without specifying the method, if any, of the consent process, or if users were well-informed about the process of data collection. Given the lack of proper documentation on the informed consent process in these studies, there is concern about the extent to which these studies adhered to legal and ethical requirements outlined in the GDPR. It is also critical to discuss whether these studies were fully compliant with the explicit consent requirements of GDPR or if they simply bypassed or minimized the importance of properly placing consent procedures. There is a need for future research to make sure explicit description of consent protocols is provided, demonstrating clear adherence to GDPR and other ethical guidelines.

##### Purpose Limitation

GDPR Article 5 focuses on how personal data can only be used for the defined purpose. However, according to De Choudhury et al [20], some AI models, in order to predict depression, reused data from social media outside the scope of the intended purpose, which is a concern of misuse of data. While the studies particularly point out such ethical issues, the studies may not have actually been compliant with the purpose limitation of GDPR or may not have discussed these without following the regulation.

The problem of purpose creep, whereby the data are used for other than the original purpose, needs more analysis. We must explore whether these AI systems in these studies were actively in line with GDPR’s restrictions on how they could use data or if the studies could operate adjacent to these restrictions, raising significant concerns of data exploitation. To ensure compliance, the AI systems will need to be designed such that strict limitations in how a purpose will be used are included, and future research should show that adherence to the principle is evidenced through clear documentation.

### Legal and Regulatory Constraints Under GDPR

#### Overview

In Europe, the GDPR, which is arguably the most sophisticated data privacy regulation in the world, places significant legal restrictions on how AI systems can process mental health data. This entails challenges to the principles of design for the operation of data-centric AI frameworks.

Pursuant to Article 9 of the GDPR, “special categories of personal data,” which includes mental health status, biometric, and genetic data, may not be processed unless one of the explicit legal bases exemplified by explicit consent, necessity for vital interest, or scientific research under adequate safeguards applies (GDPR, 2018, Article 9.2). For developers of AI systems and particularly those concerned with mental health, the limits of the law represent a unique challenge when one considers the exhaustiveness and comprehensiveness of data often collected from digital therapy bots, wearables, and social media that is assumed to be collected without informed consent and without substantive transparency mechanisms. Moreover, even without personally identifiable information, the data could be subject to regulatory carving out if it is legally identifiable by relevant ancillary information, as argued by Rocher et al [40].

In conjunction with Article 9, Article 5 provides a set of guidelines for lawful data processing, which are often contravened by AI systems integrated in mental health technologies. These are as follows:

- Legitimacy, propriety, and openness (Article 5.1.a): Patients should be informed of how AI systems use algorithms to manage their data, and more significantly, patients must be provided with genuine explanations. Unfortunately, most mental health care applications only provide vague or no explanations at all [33].
- Boundaries of purpose (Article 5.1.b): Data obtained should be confined only to the specified boundaries set by the collecting entity. There are numerous examples such as the BetterHelp case, where user data were repurposed for marketing [49].
- Data minimization (Article 5.1.c): AI-driven models may raise concerns about data minimization if they collect more data than required. However, GDPR allows data processing within the confines of explicit consent that basically ensures that only relevant data are collected and processed.
- Accuracy (Article 5.1.d) and storage limitation (Article 5.1.e): AI-performing systems should work within the scope of current and valid information. Data should be kept for only the necessary amount of time. However, there are numerous studies operating on outdated and Western datasets with no clear end point to data retention, which increases the potential for errors and abuse of information [22].

The debate above highlights a larger conflict between people’s right to data autonomy and dignity and AI’s voracious demand for huge data. They impose ethical and legal restrictions that require AI systems used in health care to be purposefully created with compliance, openness, and restraint in mind.

The European Union Artificial Intelligence Act, which came into effect in 2024, expands on existing frameworks by introducing additional legal requirements unique to AI. Because mental health AI applications have the potential to affect psychological well-being, safety, and fundamental rights, they are likely to be classified as “high risk” under the act.

#### Purpose Limitation and Commercial Exploitation

The principal limitations of purpose set forth by the GDPR restrain companies from exploiting user data without obtaining new consent, which is often ignored by companies repurposing monetizing user data. BetterHelp, a teletherapy service, for instance, is under fire for disclosing sensitive user information to advertisers like Facebook and Pinterest even when they marketed themselves as maintaining users’ confidentiality [25]. AI advertising and cloud hosting services lacking GDPR-compliant frameworks pose unchecked structural risks when partnered with third-party advertising and cloud hosting services. These incidents exemplify the risks clinicians and health care AI developers face when working with third-party advertising and cloud hosting services lacking GDPR-compliant frameworks.

#### Cross-Border Fragmentation and Equity Issues

The above cases illustrate how the extraterritorial nature of the GDPR inhibits the global deployment of AI technology. After the annulment of the Privacy Shield agreement in 2020 (Schrems II), the use of Standard Contractual Clauses or Binding Corporate Rules for Montreal-based AI companies significantly hampers research collaboration and expeditions with European Union mental health data. Gaps left by HIPAA’s limited focus on health care providers enable consumer-facing applications to exploit users. The permissive enforcement policies in low- and middle-income countries serve to capture exploitable data without legal consequences, redirecting data-capturing streams.

## Discussion

### Diagnostic Innovation

AI technologies, for instance, NLP and wearables integrated with biometric sensors, have demonstrated remarkable promise in improving the early detection of mental health conditions. However, a critical appraisal of the evidence reveals a significant imbalance in study types. The NLP-based models trained on social media data have shown approximately 89% accuracy in detecting depression and an AUC of 0.92 for suicide risk detection as identified by Gkotsis et al [6] and Coppersmith et al [21]. While these results are descriptively rich, the strength of this evidence is tempered by its retrospective nature and the disproportionate representation of NLP studies in current literature, which often lack the prospective validation required for clinical diagnostic standards.

The wearable devices like Empatica E4 have shown *F*_1_-score around 0.81 in predicting anxiety episodes in some cases [7]. These technologies are particularly beneficial in relation to scalability as well as cost-effectiveness. Further, they offer real-time tracking that has the potential to improve access to mental health care in resource-limited settings significantly. In addition, the advances in neuroimaging AI, including CNNs in schizophrenia diagnosis, have further reduced the importance of symptoms as reported by the patients [26].

Further, AI-enabled diagnostic tools consistently show high accuracy; nevertheless, when weighing these conclusions, it is vital to note that “high accuracy” in a controlled dataset does not always translate to real-world efficacy. However, there are still challenges such as the cultural bias in the NLP models. For instance, models trained on Western data often predict poorly for any non-English data as explained by Harrigian et al [22]. Additionally, wearables have problems such as sensor noise and adherence, which are major challenges in terms of real-world applications [11]. Therefore, while the volume of diagnostic evidence is high, the “certainty” of its impact in diverse clinical settings remains moderate due to these generalizability gaps. Furthermore, multimodal AI systems that combine EHRs with wearable data hold promise but require interoperability standards to function seamlessly, as highlighted by Tseng et al [28].

### Therapeutic Augmentation

AI-driven interventions such as Woebot and MindDoc provide access to personalized feedback and self-management tools, which are available 24/7. In contrast to the high volume but often retrospective NLP research, therapeutic AI research, though smaller in number of studies, often uses rigorous methods such as randomized controlled trials. These tools have demonstrated major promise with Woebot, showing an approximate 30% reduction in anxiety symptoms in clinical trials as claimed by Darcy et al [9] and Brancati et al [34].

This stratification of evidence suggests that although we have more data on AI’s potential for diagnosis (NLP), the evidence of AI’s potential for therapeutic effectiveness (chatbots) is often backed by higher-quality, prospective evidence. These interventions give the power to patients and are evolving personalized and scalable treatment beyond the clinic hours. AI chatbots like Woebot have proven to be effective when it comes to personalizing care and offering more engagement to the patients, especially those who live in urban and rural spaces. However, one important limitation is the lack of empathy displayed by such AI systems (something required in cases of high risk, such as suicidal thoughts or episodes of severe depression). Additionally, there can be hazards of algorithmic reliance, as about 25% of the patients reported their not being able to trust AI for decision-making [9].

Arya et al [35] and WHO [42] studied XAI methods such as LIME and SHAP to address this issue by providing an explanation for AI results. However, it has been reported that 40% of lower poverty clients find explanations using technical descriptions difficult [43]. Ultimately, the relative strength of evidence for therapeutic augmentation is high in terms of methodology but limited by smaller sample sizes compared to the massive datasets that are used in NLP-based screening.

### Patient Autonomy

AI tools have the potential to enhance patient autonomy greatly by giving real-time feedback, psychoeducation, and self-management tools such as MindDoc and Woebot. These interventions are essential for reducing stigma and increasing engagement by providing private access support. However, as the AI involvement in patient decision-making increases, questions about the overreliance and algorithmic paternalism also emerge. AI has proven useful in empowering patients by providing them with tools for self-management of their own mental health. However, there is a growing dependence on algorithms, and nearly 25% of patients trust the decisions that AI makes over those that clinicians make [9]. The digital literacy gap is still a big challenge. Many patients, especially those who are from lower socioeconomic strata or from the aged population, may have difficulty properly engaging with the tools with AI integration. Additionally, AI-enabled interventions without human empathy may not be as effective in building actual patient engagement. There are dangers of biases in AI, as models misdiagnose marginalized groups, for example, Black people forming words [36]. In addition, Rocher et al [40] warn that sensitive data, for example, social media and biometric inputs, to be at risk of reidentification, raising privacy concerns.

### Legal Accountability

As AI continues to be integrated into mental health care, the issue of legal accountability is a key one. Frameworks, such as GDPR and HIPAA, are making key guidelines for patient privacy. However, there are, however, concerns regarding compliance and accountability, where around 45% of mental health applications do not have HIPAA-compliant encryption [33]. There is a resulting growing consensus about the need for strong regulatory frameworks for ensuring that AI systems comply with privacy standards. However, the lack of clear definitions about liability in case of mistakes or misdiagnosis by using AI is still a major concern. AI models are often described as “black boxes”; thus, it is difficult to assign liability for errors. Moreover, no audits to verify bias and XAI tools in many systems bring questions into the topic of algorithmic fairness and transparency [48].

AI’s uses also require clear guidelines in the ethical standards aspect as a result of the problems regarding openness, reduction of biases, and involvement of multidisciplinary teams. The problem of a lack of accountability frameworks remains [5], but approaches such as federated learning [38] and differential privacy [41] promise ways of solving the data privacy and fairness problem in algorithms. The ethical framework for these systems should include mandatory testing for bias, transparency requirements on XAI, as well as compliance with privacy regulations on the order of GDPR and HIPAA.

Studies such as Jobin et al [14] and Nwosu et al [31] were concerned about the centrality of accountability for clinical AI applications. A few other studies had some measure to improve medication adherence with the use of AI tools such as AiCure [31], despite the general skepticism from therapists about “black-box” AI models [5]. A study of Kandeel et al [50] highlights the importance of interdisciplinary collaboration between clinicians, AI developers, and ethicists to ensure that these systems uphold patient autonomy, equity, and ethical standards. In the larger context of the regulatory frameworks, Afify et al [3] highlighted the role of environmental law, underlining the importance of the legal provisions in managing new emerging challenges in technology. The study was focused on AI in mental health. The study highlights the importance of adequate legislation to reduce the threat of new environmental change, which is similar to providing accountability and fairness in the application of AI in health care. Similarly, Mohamed [46] outlined the legal consequences and accountability of fully AI-based surgeries. The summation of the paper under review about the application of AI in mental health care is further represented in Table 5.

**Table 5.** Summary of artificial intelligence (AI) in mental health care.

Domain	Key applications	Benefits	Challenges	Ethical concerns
Diagnosis	NLP^a^ (social media or text analysis), wearables (biometrics), neuroimaging (fMRI^b^ or EEG^c^)	Early detection (eg, 89% accuracy for depression), scalability, low cost	Cultural bias (eg, 15% accuracy drop in non-English data), sensor noise	Privacy risks (eg, reidentification), lack of informed consent
Treatment	AI chatbots (Woebot and Wysa), predictive models, VR^d^ therapy	24/7 accessibility, personalized interventions (eg, 30% anxiety reduction)	Overreliance on AI, lack of human empathy, limited crisis handling	Algorithmic paternalism, erosion of therapeutic alliance
Patient empowerment	Mood-tracking applications (MindDoc), AI-driven psychoeducation, peer-support platforms	Enhanced self-management, reduced stigma, real-time feedback	Digital literacy gaps, algorithmic overreach	Exploitation of vulnerable users (eg, adolescents)
Privacy and security	Federated learning, differential privacy, GDPR^e^ or HIPAA^f^ compliance	Reduced reidentification risks, decentralized data training	45% applications lack encryption, data sold to third parties (eg, BetterHelp scandal)	Inequitable access, marginalized group exclusion
Accountability	Explainable AI (LIME^g^ and SHAP^h^), bias audits (IBM AI Fairness 360)	Improved trust, clinician-AI collaboration (eg, 40% error reduction)	Black-box models, inconsistent regulatory compliance	Misdiagnosis of marginalized groups (eg, racial bias in NLP)

a NLP: natural language processing.

b fMRI: functional magnetic resonance imaging.

c EEG: electroencephalogram.

d VR: virtual reality.

e GDPR: General Data Protection Regulation.

f HIPAA: Health Insurance Portability and Accountability Act.

g LIME: Local Interpretable Model-Agnostic Explanations.

h SHAP: Shapley Additive Explanations.

### Study Limitations

This review has a few limitations, which should be taken carefully into consideration while interpreting the overall study findings. First, in this study, the use of only English-language papers was considered. While this is a common approach in systematic reviews, this perhaps may have led to the exclusion of relevant studies if the paper was written in other languages. This will bring potential bias in language and will restrict the global representativeness of the evidence base (especially from non-English languages).

Second, there is a great heterogeneity across the included studies. The studies encompass several variations in study designs such as different uses of AI techniques, applications for mental health conditions, outcome measures, and considered evaluation frameworks. Due to this heterogeneity, it was not possible to carry out a meta-analytical synthesis. This review presents a broad overview; thus, the limited results of pooled statistical analysis may restrict the provision of definitive conclusions on the effective use of AI in mental health in various contexts. Consequently, the findings should be interpreted as a qualitative and thematic synthesis and not as conclusive evidence of efficacy.

Third, the included studies reported diverse and nonstandardized outcomes; therefore, comparability across studies was a major constraint. This heterogeneity in outcome definitions and ways of measurement may have impacted the consistency and generality of the conclusions drawn on issues of diagnostic accuracy, treatment effectiveness, and patient empowerment.

In addition, there was no registration of formal protocols (eg, PROSPERO) included in the review. Besides, the set systematic review guidelines were followed fully. The lack of previous registration could lead to a lower degree of transparency in a priori methodological choices and a less comprehensive ability to formally evaluate possible deviations from the planned review process.

Finally, there are legal, ethical, and regulatory interpretations of AI in mental health that also vary considerably across different regions. As a result, the findings relating to privacy, data protection, and regulatory compliance, particularly those which refer to frameworks such as GDPR and emerging AI regulations, may not be equally applicable in all legal contexts. This jurisdictional variability is a limitation in directly transferring legal and policy-related conclusions.

Despite these limitations, this review offers a structured and comprehensive synthesis of the available literature and identifies key research gaps, methodological challenges, and regulatory considerations that can inform future empirical research and policy development in the context of AI-enabled mental health care.

### Review Recommendations

The study, after reviewing several papers, offers the following recommendations:

- AI models should be trained on multilingual and diverse datasets, in a way that would make them culture-independent. This will eliminate prejudice and guarantee accuracy in mental health diagnoses across the world.
- Enact strong ethical frameworks that extend into the areas of data privacy and patient-informed consent and algorithm transparency. Implement ideas of existing regulatory frameworks such as GDPR and HIPAA for data protection of patients.
- Participatory frameworks to develop region-specific AI models that incorporate local patients and practitioners.
- Work on differential privacy (adding statistical noise to datasets) or federated learning (training AI on decentralized datasets without data sharing), adhering to GDPR regulations.
- Promoting international data governance agreements to expedite international health research while upholding local customs.
- Implement XAI techniques like LIME and SHAP in a system that would provide explanations for AI recommendations in a way that is understandable. Such transparency will encourage trust between patients and clinicians with AI recommendations.
- Design AI-supported models for additive decision support for clinicians instead of replacing human judgment. Using hybrid models that enhance accuracy in diagnosis and treatment planning while preserving the human touch in health care.
- As part of routine checking, regular audits should be carried out using tools like IBM’s AI Fairness 360 to identify and rectify biases within AI algorithms.

**Figure 2.**
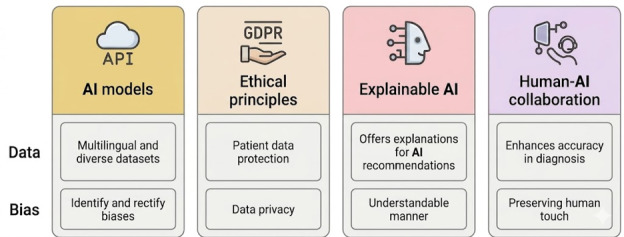
Major review outcome of AI applications in mental health based upon data and bias for proper applications. AI: artificial intelligence; API: application programming interface; GDPR: General Data Protection Regulation.

Overall, based upon a critical and systematic review, comparison, and coherence of studies, this study develops a review of findings as illustrated in Figure 2.

### Conclusions

The widespread adoption of AI in mental health represents a significant technological evolution. Besides, its role as a paradigm shift remains a future potential rather than a current clinical reality. In addressing mental health care needs, AI demonstrates potential as a supportive tool for screening, monitoring, and digitally delivered interventions. Nonetheless, current evidence remains heterogeneous and context-dependent, with the strength of conclusions often limited by the varying risk of bias across included studies. Claims regarding cost-effectiveness, cultural sensitivity, and large-scale clinical impact require further empirical validation through robust, longitudinal, and comparative studies that prioritize high-certainty evidence. AI-enabled devices, such as NLP models, wearables equipped with biometric sensors, and neuroimaging procedures, have been found to be useful in the diagnosis of mental health disorders such as depression, anxiety, and schizophrenia. NLP models from social media, for example, have been found to be extremely accurate at the detection of suicide and depression, although the results are highly underrepresented elsewhere in terms of clinical trials and are instead collected retrospectively. Wearables such as Expatica’s E4 have been found helpful for real-time physiology monitoring.

As these technological advancements are significant, the weighting of their clinical impact must be balanced against the prevalence of moderate-to-high risk of bias in the current literature. The use of AI for the treatment of mental health has high ethical, technical, and practical implications. Data privacy concerns about algorithmic bias and the need for culturally sensitive devices bring home the difficulty of integration of AI in clinical practice. Use-based dependency upon AI devices has raised the problem of patient agency and algorithmic dependency, particularly if a significant judgment is being left in the hands of chatbots. These concerns should be addressed through adequate ethical guidelines, stratification of evidence courtesy of its real-world applicability, involvement of stakeholders, and constant evaluation of AI technology for the greatest potential of AI in mental health treatment. All these will allow AI as an aid for human expertise and not as a substitute for human expertise, while ensuring patient confidence and safety, and improving the quality and access of mental health services. Additionally, AI has the potential to revolutionize mental health care, though the necessary regulatory frameworks, such as GDPR and HIPAA, have to have a complete integration with the development and deployment of these systems. For AI to be effective and ethical in the treatment of mental health, a continued effort of research and development involves the importance of a rigorous analytical framework, regulatory compliance, transparency, and patient autonomy. By addressing these challenges and focusing on high-quality, prospectively validated evidence, AI can be implemented in a manner that ensures data privacy, reduces algorithmic bias, and promotes trust between patients and health care providers.

### Future Research Directions

The future research directions are as follows:

- Perform longitudinal research in order to look into the long-term effectiveness of AI interventions for mental health. Such research will inform us about the effectiveness of AI interventions and what harm can be done through long-term use of AI.
- Research on combining various sources such as EHRs, wearables, and patient-reported results in a single overall mental health assessment AI system.
- Research into the use of AI for crisis intervention would have to account for the strengths and limitations of AI technology in the context of acute mental health crisis situations. Research would need to be conducted in order to ensure that AI technology possesses the skills to identify and respond in an emergency situation and steer the individuals toward the crisis human support when required.
- Evaluate various patient engagement strategies for AI-enabled solutions in patient populations whose digital literacy levels span across a range of domains. An understanding of user behavior will inform the development of less complex, user-friendly AI applications.
- Work together with policymakers toward drafting policies that enable proper and ethical use of AI in mental health service provision. Such policies should touch upon matters such as liability, standardization, and reimbursement for AI service provision.
- In the future, studies need to cover explicit documentation of GDPR and HIPAA compliance. This may include a detailed account of consent mechanisms, data anonymization techniques, and the implementation of privacy by design principles in AI development.

## Supplementary material

10.2196/84305 Checklist 1 PRISMA checklist.

## Abbreviations

AI: artificial intelligence
AUC: area under the curve
CBT: cognitive behavioral therapy
CNN: convolutional neural network
EAI: explainable artificial intelligence
EHR: electronic health record
FDA: Food and Drug Administration
GDPR: General Data Protection Regulation
HIPAA: Health Insurance Portability and Accountability Act
HRV: heart rate variability
LIME: Local Interpretable Model-Agnostic Explanations
NLP: natural language processing
PRISMA: Preferred Reporting Items for Systematic Reviews and Meta-Analyses
SaMD: Software as a Medical Device
SHAP: Shapley Additive Explanations
WHO: World Health Organization
